# Circadian rhythms are more resilient to pacemaker neuron disruption in female *Drosophila*

**DOI:** 10.1371/journal.pbio.3003146

**Published:** 2025-05-06

**Authors:** Aishwarya Ramakrishnan Iyer, Eva Scholz-Carlson, Evardra Bell, Grace Biondi, Shlesha Richhariya, Maria P. Fernandez

**Affiliations:** 1 Department of Biology, Indiana University Bloomington, Bloomington, Indiana, United States of America; 2 Department of Neuroscience and Behavior, Barnard College, New York City, New York, United States of America; 3 HHMI, Brandeis University, Waltham, Massachusetts, United States of America; Washington University, St. Louis, MO 63110, UNITED STATES OF AMERICA

## Abstract

The circadian system regulates the timing of multiple molecular, physiological, metabolic, and behavioral phenomena. In *Drosophila,* as in other species, most of the research on how the timekeeping system in the brain controls the timing of behavioral outputs has been conducted in males, or sex has not been included as a biological variable. A critical set of circadian pacemaker neurons in *Drosophila* release the neuropeptide pigment-dispersing factor (PDF), which functions as a key output factor in the network with complex effects on other clock neurons. Lack of *Pdf* or its receptor, *PdfR,* results in most flies displaying arrhythmicity in activity–rest cycles under constant conditions. However, our results show that female circadian rhythms are less affected by mutations in both *Pdf* and *PdfR*. CRISPR-Cas9-mediated mutagenesis of *Pdf,* specifically in ventral lateral neurons (LN_v_s), also has a greater effect on male rhythms. We tested the influence of M-cells on the circadian network and showed that speeding up the molecular clock specifically in M-cells led to sexually dimorphic phenotypes, with a more pronounced effect on male rhythmic behavior. Our results suggest that the female circadian system is more resilient to manipulations of M-cells and the PDF pathway, suggesting that circadian timekeeping is more distributed across the clock neuron network in females.

## Abstract 2

Circadian pacemaker neurons regulate the speed of the clock in the Drosophila brain, but the effect of sex as a biological variable remains unclear. This study reveals that female Drosophila are more resilient to disruptions in circadian rhythms caused by mutations in the PDF pathway and alterations in pacemaker cell clock speed.

## Introduction

Differences in neuronal circadian timekeeping between sexes remain relatively unexplored, despite the expanding body of research highlighting the influence of sex on the mechanisms underlying neuronal control of behavior [1]. In mammals, steroid hormones display daily, clock-driven changes in abundance, and while these sex hormones are not required to maintain rhythms, they differentially influence the amplitude of activity behavior between the sexes [1,2]. Furthermore, structural and functional sex differences have been observed in brain areas that receive direct input from the brain’s circadian timekeeping center [2,3]. Research in humans has also revealed significant sexual dimorphism: men tend to have lower-amplitude endogenous rhythms than women [4], are less resilient to nocturnal sleep disruptions, and spend less time asleep [5].

In mammals, the main circadian pacemaker resides in the suprachiasmatic nuclei (SCN), which in mice consist of a network of ~ 20,000 neurons (reviewed in [6]). The *Drosophila* circadian clock network has ~ 240 neurons and is the functional equivalent of the mammalian SCN [7–9] (reviewed in [10]). Each circadian clock neuron has an intracellular molecular timekeeping mechanism based on a transcriptional–translational feedback loop: the genes *Clock* (*Clk*) and *cycle* (*cyc*) promote rhythmic transcription of several key genes, including *period* (*per*) *and timeless (tim*), which build up daily and inhibit their own transcription [11]. Multiple kinases that act on components of these clock proteins and can affect the pace of the molecular clock have been identified. One such kinase is *doubletime* (DBT), which binds to and phosphorylates PER, regulating its nuclear accumulation [12,13].

The fly clock network consists of *lateral neurons* (*LNs*), which include ventrolateral (LN_v_), dorsolateral (LN_d_), and lateral posterior neurons (LPNs), as well as three groups of *dorsal neurons* (*DN1, DN2, and DN3*), some of which can be further subdivided [8,14–17]. The ventral and dorsal LNs are sufficient to produce the normal endogenous bimodal rhythm of sleep and activity [18,19]. The small ventral lateral neurons (s-LN_v_s) are usually referred to as morning cells (M-cells) since they control the morning peak of activity under light–dark cycles (LDs). These cells are also essential for maintaining rhythmicity under free-running conditions [20,21]. The evening peak is controlled by the LN_d_s and a *Pdf*-negative LN_v_, the 5th LN_v_ (E-cells) [18,22,23]. Some dorsal neurons (DNs) also contribute to the timing and amount of sleep via the modulation of M and E cells [24–27].

The release of the circadian neuropeptide pigment-dispersing factor (PDF) by s-LN_v_s is essential for endogenous circadian timekeeping. A *Pdf* null mutation, *Pdf*^*01*^, results in a substantial fraction of arrhythmic flies [20], desynchronization of molecular oscillations [28,29], and phase changes in the electrical activity of some clock clusters, most notably the LN_d_s [30]. Loss of *PdfR* also leads to loss of behavioral rhythms [31–33]. Interestingly, PDF and PDFR also regulate behaviors that are sex-specific or sexually dimorphic. Rival-induced long mating durations require PDF expression in s-LN_v_s, PDFR expression in a subset of LN_d_s, and NPF expression in LN_d_s [34]. PDF controls rhythms in the sexually dimorphic pheromone profiles produced by oenocytes [35] and is involved in long-term mating suppression in males [36]. Both PDF and PDFR contribute to geotactic behaviors [31], and the phenotypes of *Pdf*^01^ mutants are sexually dimorphic, with males showing a more extreme negative geotaxis phenotype [37].

Sexual dimorphism in *Drosophila* sleep/wake cycles has been studied mostly under LD cycles. Males exhibit lower levels of activity and more sleep during the light phase [38–40]. This increase in midday sleep is due to the activity of a subset of sleep-promoting DN1s, which are more active in males [38] and receive input from the male-specific P1 neurons that control male courtship [39]. Unlike studies on circadian rhythms, *Drosophila* sleep research often involves only females. Males also have an earlier and more pronounced morning peak and a larger phase angle between the morning peak and the evening peak [41]. Under conditions of constant darkness and temperature (DD), males of several wild-type strains have a small but significant reduction in the free-running period (FRP) relative to females of the same strain [41]. Moreover, males are more likely to retain a bimodal activity pattern in DD [41]. A recent transcriptomic analysis of *fruitless* (*fru*)-expressing neurons revealed clusters that are enriched for circadian clock genes [42]. A previous study reported that DN1s express the male-specific Fru^M^ protein [43] and that the number of cells in the DN1_p_s cluster is sexually dimorphic [44]. In addition, the E3 subset of LN_d_s has been shown to be dimorphic in its expression of the neuropeptide NPF [45,46].

Given the sexually dimorphic roles of neuropeptides, including PDF, in other behaviors [47], we asked whether females were similarly affected by manipulations of the *Pdf/PdfR* pathway. We found that female circadian rhythms are less affected by null mutations in both *Pdf* and *PdfR* and that similar effects are observed via CRISPR-Cas9-mediated *Pdf* mutagenesis, specifically in the LN_v_s. Moreover, speeding up the molecular clock in the LN_v_s via expression of *DBT*^*s*^ leads to an advance of the morning peak in males but not in females, and the pace of the FRP of activity is significantly shortened only in males. Taken together, our results show that the female circadian system is more resilient to manipulations in the PDF pathway and suggest that *Pdf*+ neurons play a more dominant role in the male than in the female circadian network.

## Results

### Mutations in PDF and PDFR lead to sexually dimorphic phenotypes

**Fig 1:**
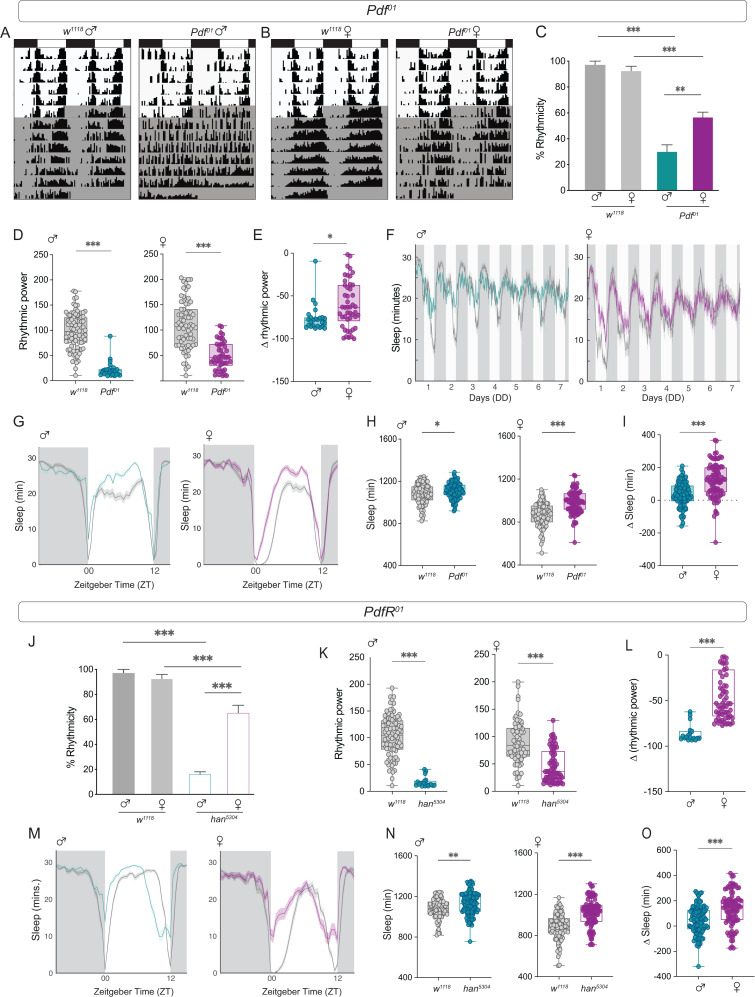
Mutations in *Pdf* and *PdfR* lead to sexually dimorphic phenotypes. **(A)** Representative double-plotted actograms of *w*^*1118*^ (left) and *Pdf*^*01*^ (right) male flies subjected to 6 days of LD followed by seven days of DD. **(B)** Representative actograms of *w*^*1118*^ (left) and *Pdf*^*01*^ (right) female flies subjected to six days of LD followed by seven days of DD. **(C)** Percentages of rhythmic flies are plotted for control (*w*^*1118*^) and *Pdf*^*01*^ males (*n* = 86 (*w*^*1118*^), *n* = 81 (*Pdf*^*01*^)) and females (right, *n* = 75 (*w*^*1118*^), *n*  = 101 (*Pdf*^*01*^)). The error bars represent the SEM values plotted across three replicate experiments. **(D)** Rhythmic power of control (*w*^*1118*^) and *Pdf*^*01*^ males and females were calculated using the Chi-squared Periodogram. **(E)** The differences in rhythmic power between experimental males and females and their respective controls are plotted. **(F)** Average sleep plots of flies over seven days in DD are plotted for male and female control (*w*^*1118*^) and experimental (*Pdf*^*01*^) flies. The controls are plotted in gray, and the experimental males and females are plotted in blue and magenta, respectively. **(G)** Average sleep plots under LD 12:12 for the control (*w*^*1118*^) and experimental (*Pdf*^*01*^) groups are plotted for males (left) and females (right). The plots are averaged over flies and days for a period of three days under LD 12:12. The controls are plotted in gray, and the experimental males and females are plotted in blue and magenta, respectively. **(H)** Total sleep values under LD conditions are plotted for male (left) and female (right) control (*w*^*1118*^) and experimental (*Pdf*^*01*^) flies. **(I)** The differences in total LD sleep values between experimental males and females and their respective controls are plotted. **(J)** Percentage of rhythmic flies are plotted for control (*w*^*1118*^) and *han*^*5304*^ males (*n* = 86 (*w*^*1118*^), *n* = 115 (*han*^*5304*^)) and females (*n* = 74 (*w*^*1118*^), *n* = 94 (*han*^*5304*^)) **(K)** Rhythmic power of the control (*w*^*1118*^) and *han*^*5304*^ males and females calculated using the Chi-squared periodogram are plotted. **(L)** The differences in rhythmic power between experimental males and females and their respective controls are plotted. **(M)** Average sleep plots under LD 12:12 for control (*w*^*1118*^) and experimental (*han*^*5304*^) flies are plotted for males (left) and females (right). The plots are averaged over flies and days for a period of three days under LD 12:12. The controls are plotted in gray, and the experimental males and females are plotted in blue and magenta, respectively. **(N)** Total sleep values under LD conditions are plotted for male (left) and female (right) control (*w*^*1118*^) and experimental (*han*^*5304*^) flies. **(O)** The differences in total LD sleep values between experimental males and females and their respective controls are plotted. Statistical comparisons were performed between the control and experimental flies of both sexes using a Mann–Whitney *U* test and percentage of rhythmic flies were compared using Fisher’s exact test. The box plots extend from the 25th to 75th percentile, with whiskers extending from the smallest to the largest value, and each point represents data from a single fly. Combined data from at least three replicate experiments are plotted. *p < 0.05, **p < 0.01, ***p < 0.001.

A null mutation in *Pdf* results in pronounced behavioral phenotypes in *Drosophila* males [20]. We assayed the locomotor activity rhythms of *Pdf*^*01*^ females under free-running conditions (DD) and found that a large proportion of the experimental females were still rhythmic (Fig 1A–1C). The rhythmic power of experimental flies was significantly reduced in both sexes (Fig 1D), but the effect was less pronounced in females (Fig 1E), suggesting that *Pdf*^*01*^ females have more consolidated rhythms than *Pdf*^*01*^ males. We employed virgin females, as female rhythm strength has been shown to be significantly reduced after mating [48]. Mutant females that were rhythmic had a slightly, but significantly, shorter FRP than the controls (Table 1). This phenotype was not observed in experimental males (Table 1), consistent with a recent study [49]. Sleep cycles under DD also appeared to be more consolidated in females (Fig 1F). *Pdf* mutants have increased sleep, and this effect is mediated by PDF acting on the LN_v_s themselves [50]. We found that both sexes show an increase in total sleep in LD, but the effect was more pronounced in females (Fig 1G–1I). While the increase in sleep in males was most prominent at midday, females exhibited increased sleep throughout most of the light phase (Fig 1G). We are employing a *Pdf*^*01*^ mutant in *w*^*1118*^ background, and neither the advanced evening peak nor the short FRP of the *Pdf* null mutant are consistently observed. The lack of a short FRP in rhythmic *Pdf*^*01*^ mutant flies in this genetic background is consistent with a previous study [49].

**Table 1:** Table representing the *n*, % rhythmicity, phase of the E peak, free-running period, and rhythmic power of *w*^*1118*^ and *Pdf*^*01*^ males and females.

*Pdf* ^*01*^					
*Genotype*	*n*	% Rhythmicity ± SEM	E-peak phase ± SEM	Free-running period ± SEM	Rhythmic power ± SEM
*w1118 (male)*	86	97.3 ± 2.66	11.92 ± 0.07	23.8 ± 0.04	97.8 ± 3.88
*Pdf01 (male)*	*81*	30.3 ± 5.05^**#^	11.72 ± 0.07	23.72 ± 0.13	22.1 ± 3.24^***#^
*w1118 (female)*	*75*	92.8 ± 3.27	11.71 ± 0.07	24.1 ± 0.04	109.9 ± 5.63
*Pdf01 (female)*	*101*	56.9 ± 3.63^**#^	11.85 ± 0.08	23.9 ± 0.09^*^	49.63 ± 4^***#^

* indicates that the experimental genotypes are significantly different from their respective control flies of the same sex.

# indicates that experimental males and females are significantly different from each other.

* *p* < 0.05,

** *p* < 0.01,

*** *p* < 0.001.

To rule out the presence of remnant PDF expression in *Pdf*^*01*^ females, we stained the brains of control and experimental males and females with an anti-PDF antibody. We did not observe any traces of PDF in experimental flies of either sex, even with increased laser intensity (S1A Fig). PDF accumulates rhythmically in the dorsal termini of the s-LN_v_ projections in a time-of-day-dependent manner both in LD and DD [20,21]. To determine if there were differences in the amplitude of PDF cycling between the sexes in a wild-type background (Canton-S), we dissected control males and females on the third day under DD at 6 time points over a 24-h cycle. Using a COSINOR-based curve fitting method [51], we found that both males and females have clear 24-h rhythms in PDF cycling in their dorsal projections, with no significant sex differences in amplitude (S1B and S1C Fig and Table 2).

**Table 2:** Cosinor analysis parameters for *Canton-S* males and females.

Cosinor parameters for PDF cycling in s-LNv dorsal termini					
Genotype	p value	PR	Mesor ± s.e.	Amplitude ± s.e.	Phase ± s.e.
*Canton-S* (male)	2.02e − 06	42.1	17.15 ± 2.67	22.38 ± 3.79	−4.71 ± 9.64
*Canton-S* (female)	7.33e − 07	37.554	14.59 ± 2.9	24.47 ± 4.07	−19.13 ± 9.65

Next, we asked whether the effects of a *Pdf* receptor mutation (*PdfR*) on activity and sleep were also sexually dimorphic. The expression of PDFR, a GPCR, can be detected in most clock neurons, with the exception of 3 LN_d_s, half DN1ps, and some DN3s [52], which coincides with *Cryptochrome* expression in clock neurons [52]. The *han*^*5304*^ mutant is a *PdfR* hypomorph and exhibits *Pdf*^*01*^-like behavioral phenotypes under both LD 12:12 and DD [31–33]. Under DD, both *Han* males and females showed a significant reduction in rhythmicity compared with the controls, but there was a greater proportion of rhythmic females (~65%) than males (~16%) (Fig 1J). The FRP of the experimental flies was significantly shorter for both sexes (Table 3), as reported previously for males. Rhythmic power was significantly lower than that of the controls for both *han*^*5304*^ males and females (Fig 1K), but the effect was more pronounced in males, suggesting that females have more consolidated rhythms (Fig 1L). Similar to the effect of the *Pdf* mutation, *han*^*5304*^ flies showed significantly higher levels of LD sleep than controls, and this effect was also more pronounced in females (Fig 1M–1O). Taken together, these results suggest that female circadian rhythms are less affected by the loss of both *Pdf* and *PdfR*. However, the LD sleep phenotypes were more pronounced in females.

**Table 3:** Table representing the *n*, % rhythmicity, phase of the E-peak, free-running period, and rhythmic power of *w*^*1118*^ and *PdfR*^*01*^ males and females.

*PdfR* ^*01*^					
*Genotype*	*n*	% Rhythmicity ± SEM	E-peak phase ± SEM	Free-running period ± SEM	Rhythmic power ± SEM
*w1118 (male)*	*86*	97.3 ± 2.66	11.68 ± 0.07	23.8 ± 0.03	103.1 ± 4.15
*PdfR01 (male)*	*115*	16.4 ± 1.64^***#^	10.33 ± 0.04	21.8 ± 0.12^***#^	17.46 ± 2.28^***#^
*w1118 (female).*	*74*	92.7 ± 3.25	11.45 ± 0.07	24.1 ± 0.05	89.13 ± 4.75
*PdfR01 (female)*	*94*	65.4 ± 5.8^***#^	10.36 ± 0.07	22.4 ± 0.07^***#^	47.3 ± 3.5^***#^

* indicates that the experimental genotypes are significantly different from their respective control flies of the same sex. # indicates that experimental males and females are significantly different from each other. *p < 0.05, **p < 0.01, ***p < 0.001.

### CRISPR-Cas9-mediated *Pdf* mutagenesis has more pronounced effects on male behavior

In the *Pdf* null mutant, background effects could contribute to the sexual dimorphism observed in behavioral rhythms. We therefore employed a tissue-specific CRISPR-Cas9-mediated knockout of *Pdf* in both males and females, as described in a recent study that focused on males [53]. To assess the efficiency of the manipulation, we stained for PDF in flies that constitutively expressed *Pdf* gRNA and Cas9 in *Pdf*+ neurons. This experiment was conducted at 28 °C, as this temperature was more effective at mimicking the behavioral phenotypes of the *Pdf*^01^ mutant males.

**Fig 2:**
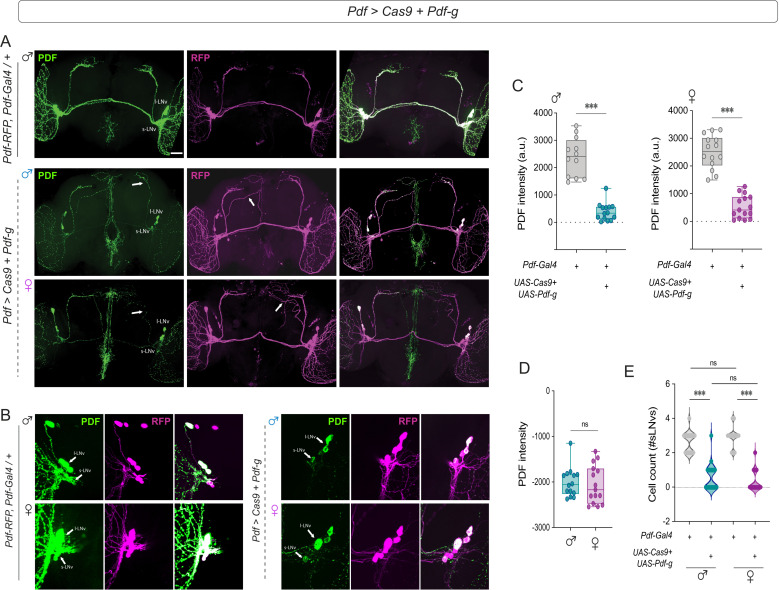
Tissue-specific CRISPR-mediated *Pdf* manipulation leads to a reduction in PDF levels and the misrouting of s-LN_v_ dorsal termini in both sexes. **(A)** Representative confocal images of control (*Pdf-RFP, Pdf-Gal4; tub-Gal80*^*ts*^) (top) and experimental (*Pdf-RFP, Pdf-Gal4; tub-Gal80*^*ts *^*> Cas9; Pdf-g*) (middle, males; bottom, females) flies stained with RFP and PDF antibodies. Experimental flies show a significant reduction in PDF levels in the s-LN_v_s (white arrows, PDF channel) and misrouting of the s-LN_v_ dorsal termini (white arrows, RFP channel). **(B) (left)** Representative confocal images of the small and large LN_v_s of control (*Pdf-RFP, Pdf-Gal4; tub-Gal80*^*ts*^) (males, top) and (females, bottom), **(right)** Representative confocal images of the small and large LN_v_s of experimental (*Pdf-RFP, Pdf-Gal4; tub-Gal80*^*ts *^*> Cas9; Pdf-g*) (males, top) and (females, bottom) brains stained with RFP and PDF antibodies. **(C)** Quantification of PDF levels from s-LN_v_ cell bodies in control (*Pdf-RFP, Pdf-Gal4; tub-Gal80*^*ts*^) and experimental (*Pdf-RFP, Pdf-Gal4; tub-Gal80*^*ts *^*> Cas9; Pdf-g*) flies are plotted for males (left) and females (right). *n* > 12 brains for all genotypes. **(D)** Differences in the PDF intensity values of experimental males and females from their respective parental controls. **(E)** Number of PDF-positive s-LN_v_s in each brain are plotted for control (*Pdf-RFP, Pdf-Gal4; tub-Gal80*^*ts*^) and experimental (*Pdf-RFP, Pdf-Gal4; tub-Gal80*^*ts *^*> Cas9; Pdf-g*) males and females. *n* > 13 brains for all genotypes. Statistical comparisons were performed between the control and experimental flies of both sexes using the Mann–Whitney *U* test. The box plots extend from the 25th to 75th percentile, with whiskers extending from the smallest to the largest value, and each point represents data from a single fly. Combined data from at least two replicate experiments are plotted. *p < 0.05, **p < 0.01, ***p < 0.001. Scale bars = 50 μm.

PDF was reduced in the s-LN_v_s in both sexes (Fig 2A–2C), although in most experimental brains, we noted faint staining in the dorsal projections of at least one s-LN_v_ in at least one brain hemisphere (Fig 2A). We quantified PDF intensity in the cell bodies of the s-LN_v_s and found that the signal intensity and the number of cells were reduced in both sexes in a similar manner (Fig 2B–2E). PDF expression within the large LN_v_s was less affected and could be detected in 2–3 l-LNv cell bodies in most brains (Fig 2A). In addition to behavioral phenotypes, *Pdf*^01^ mutation leads to pronounced misrouting of s-LN_v_ projections in male flies [54]. We employed a transgene expressing a red fluorescent protein under the *Pdf* regulatory sequence [55] and observed faint projections occasionally defasciculating from the main bundle in one or both hemispheres in *Pdf *> *Pdf*-g; *Cas9* flies of both sexes (Fig 2A, middle panels). To determine whether driver strength was similar between males and females, we analyzed nuclear signal levels in the s-LN_v_s of male and female *Pdf > nls-mCherry* flies and found no significant sex differences (S1D Fig). However, subtle sex differences in driver strength may be undetectable due to the constitutively high expression levels of the Gal4/UAS system.

**Fig 3:**
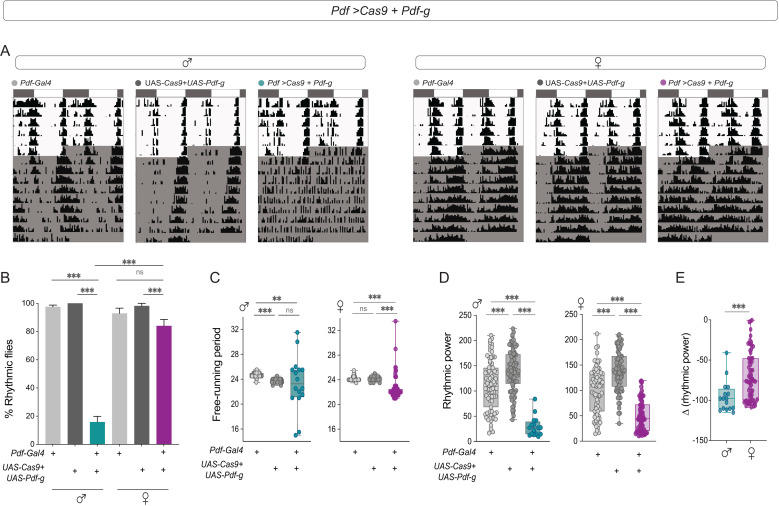
CRISPR-Cas9-mediated *Pdf* mutagenesis has more pronounced effects on male behavior. **(A)** Representative actograms of control (*Pdf-RFP, Pdf-Gal4; tub-Gal80*^*ts*^) and *(UAS Cas9; Pdf-g)* and experimental (*Pdf-RFP, Pdf-Gal4; tub-Gal80*^*ts *^*> Cas9; Pdf-g*) males (left) and females (right) are plotted for six days of LD followed by nine days of DD. **(B)** Percentage of rhythmic flies are plotted for control (*Pdf-RFP, Pdf-Gal4; tub-Gal80*^*ts*^*) and (UAS Cas9; Pdf-g)* and experimental (*Pdf-RFP, Pdf-Gal4; tub-Gal80*^*ts *^*> Cas9; Pdf-g)* males (*n* = 85 (*Pdf-RFP, Pdf-Gal4; tub-Gal80*^*ts*^), *n* = 89 (*UAS Cas9; Pdf-g*), *n* = 106 (*Pdf-RFP, Pdf-Gal4; tub-Gal80*^*ts *^*> Cas9; Pdf-g*)) and females (*n* = 79 (*Pdf-RFP, Pdf-Gal4; tub-Gal80*^*ts*^), *n* = 78 (*UAS Cas9; Pdf-g*), *n *= 85 (*Pdf-RFP, Pdf-Gal4; tub-Gal80*^*ts *^*> Cas9; Pdf-g)).* The error bars represent SEM values plotted across three replicate experiments. **(C)** Free-running periods of control (*Pdf-RFP, Pdf-Gal4; tub-Gal80*^*ts*^ *and UAS Cas9; Pdf-g)* and experimental (*Pdf-RFP, Pdf-Gal4; tub-Gal80*^*ts *^*> Cas9; Pdf-g)* males (left) and females (right) calculated via the chi-squared periodogram are plotted. **(D)** Rhythmic power of control (*Pdf-RFP, Pdf-Gal4; tub-Gal80*^*ts*^ *and UAS Cas9; Pdf-g)* and experimental (*Pdf-RFP, Pdf-Gal4; tub-Gal80*^*ts *^*> Cas9; Pdf-g)* males (left) and females (right) calculated via the chi-squared periodogram are plotted. **(E)** The differences in rhythmic power of experimental males and females from their respective controls are plotted. Flies were kept at 28 °C throughout development, and as adults, experiments were conducted at 28 °C. Statistical comparisons were performed between the control and experimental flies of both sexes using the Kruskal–Wallis test followed by Dunn’s multiple comparisons test for panels B–D and Mann–Whitney *U* test for Fig 3E. Percentage of rhythmic flies were compared using Fisher’s exact test. The box plots extend from the 25th to 75th percentile, with whiskers extending from the smallest to the largest value, and each point represents data from a single fly. Combined data from at least three replicate experiments are plotted. **p < 0.01, ***p < 0.001.

We analyzed activity–rest rhythms in *Pdf *> *Pdf*-g; *Cas9* flies and found that while experimental males were largely arrhythmic, the percentage of rhythmic experimental females was not different from the controls (Fig 3A and 3B). The FRP of the experimental males was not significantly different from that of the controls, but a wide range of periods were observed. Compared with parental controls, *Pdf *> *Pdf*-g; *Cas9* females had significantly shorter FRPs (Fig 3C). The rhythmic power was significantly lower in experimental flies of both sexes (Fig 3D), but the effect was less pronounced in females (Fig 3E). Sleep under LD was not affected (S2A and S2B Fig), whereas sleep under DD was similarly increased in males and females, both for several days under DD and for DD1 only (S2C–S2F Fig). We also calculated the activity/waking minute for control and experimental flies and found that there are no significant differences in the waking activity for experimental flies of both sexes (S2G Fig).

We next restricted the CRISPR mutagenesis of *Pdf* to the small LN_v_s via a specific driver from the split-Gal4 collection generated by the Rubin Laboratory (*SS00681*-Gal4). *Pdf* knockdown in the s-LNv resulted in most males being arrhythmic (~30% rhythmicity), whereas the experimental females were ~57% rhythmic (S3A and S3E Fig and S1 Table). The percentage of rhythmic flies was significantly lower than that of both controls for both experimental males and females, but females were significantly more rhythmic (S3A Fig). The FRP of the experimental males and females was shorter than that of their respective control flies (S3B Fig). The rhythmic power of experimental males and females was lower than that of their respective controls (S3C Fig), but the effect was less pronounced in females (S3D Fig). This suggests that PDF from the s-LNv is important for the behavioral and sex-specific differences observed in *Pdf*^*01*^ mutants.

### Speeding up the M-cell clock results in a more effective period shortening in males

**Fig 4:**
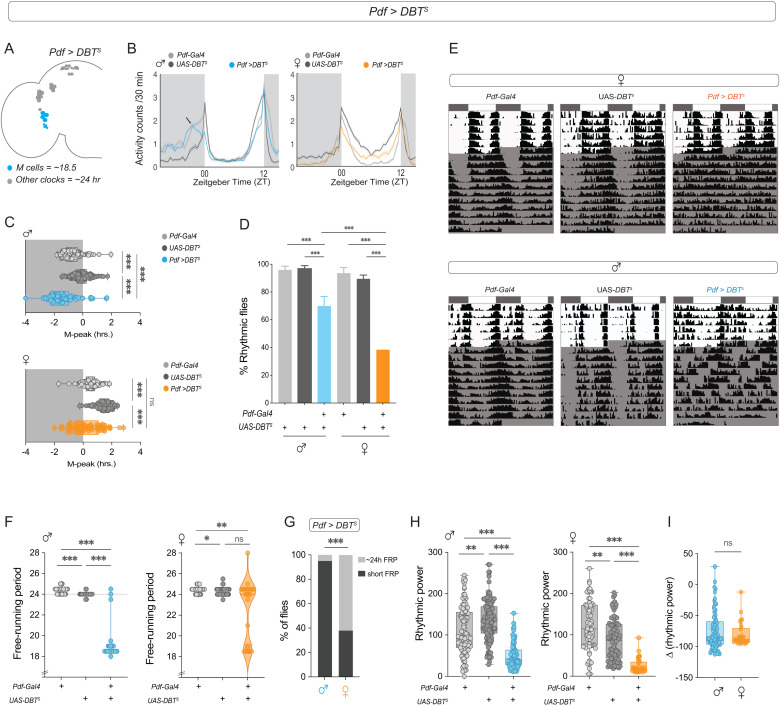
Speeding up the clock in M-cells leads to sexually dimorphic phenotypes. **(A)** Depiction of the adult *Drosophila* brain hemisphere indicating the clock cell subsets (colored) having a faster running molecular clock. **(B)** Average activity plots of control (*Pdf-Gal4*) and (UAS-*DBT*^*s*^) and experimental (*Pdf > DBT*^*s*^) flies are plotted for males (left) and females (right). The plots are averaged over flies and days for a period of three days under LD 12:12. **(C)** The phase of the morning peak of activity under LD 12:12 is plotted for control (*Pdf-Gal4*), (UAS-*DBT*^*s*^) and experimental (*Pdf > DBT*^*s*^) males (left, *n* = 116 (*Pdf-Gal4*), *n *= 120 (UAS-*DBT*^*s*^), *n* = 88 (*Pdf > DBT*^*s*^)) and females (right, *n* = 85 (*Pdf-Gal4*), *n* = 109 (UAS-*DBT*^*s*^), *n* = 73 (*Pdf > DBT*^*s*^)). **(D)** Percentages of rhythmic flies are plotted for controls (*Pdf-Gal4), (UAS-DBT*^*s*^*),* and experimental (*Pdf > DBT*^*s*^) males (*n* = 90 (*Pdf-Gal4*), *n* = 108 (*UAS-DBT*^*s*^), *n* = 93 (*Pdf >*^* *^*DBT*^*s*^) and females (*n* = 66 (*Pdf-Gal4*), *n* = 83 (*UAS-DBT*^*s*^), *n* = 70 (*Pdf*^* *^*> DBT*^*s*^*).* The error bars represent the SEM values plotted across three replicate experiments. **(E)** Representative actograms of controls (*Pdf-Gal4* and UAS-*DBT*^*s*^) and experimental (*Pdf > DBT*^*s*^) females (top) and males (bottom) plotted for five days of LD followed by 10 days of DD. **(F)** Free-running periods of control (*Pdf-Gal4), (UAS-DBT*^*s*^) and experimental (*Pdf*^* *^*> DBT*^*s*^) males (left) and females (right) calculated via the chi-squared periodogram are plotted. **(G)** Percentage of flies having a short (18–21.5 h) and normal (23–25 h) free-running periods are plotted for experimental (*Pdf > dBTs*) males and females **(H)** Rhythmic power of control (*Pdf-Gal4), (UAS-DBT*^*s*^) and experimental (*Pdf*^* *^*> DBT*^*s*^) males (left) and females (right) calculated using the Chi-squared periodogram are plotted. **(I)** The differences in rhythmic power between experimental males and females and their respective controls are plotted. Statistical comparisons were performed between the control and experimental flies of both sexes using the Kruskal–Wallis test followed by Dunn’s multiple comparisons test for panels C, F, and H and Mann–Whitney *U* test for Fig 4I. Percentage of rhythmic flies were compared using Fisher’s exact test. The box plots extend from the 25th to 75th percentile, with whiskers extending from the smallest to the largest value, and each point represents data from a single fly. Combined data from at least three replicate experiments are plotted. *p < 0.05, **p < 0.01, ***p < 0.001.

Next, we sought to determine if the influence of the *Pdf*-releasing cells themselves was sexually dimorphic. While PDF is released from both large and small LN_v_s, only s-LN_v_s (Morning cells) play key roles in regulating free-running rhythm properties [18]. In males, manipulations that change the pace of the clock specifically in the LN_v_s result in changes in the phase of the morning peak of activity and in FRP [22,56]. We expressed the *doubletime* ‘short’ (*DBT*^s^) allele [57] under the *Pdf-Gal4* driver (Fig 4A) and analyzed the effects on behavior in both sexes. We found that *Pdf* > *DBT*^s^ males, but not females, have an advanced phase of the morning peak of activity (M-peak, Fig 4B and 4C). This suggests that under LD cycles, the M-oscillator is more effective at setting the phase of male than female behavior.

Under free-running conditions, both *Pdf* > *DBT*^s^ male and female flies showed a significantly lower percentage of rhythmic flies than their controls (Fig 4D), but there were fewer rhythmic females (~40%) than males (~65%) (Fig 4D and 4E). The FRP of most *Pdf* > *DBT*^s^ males was ~ 18.5 h (Fig 4F), whereas the *Pdf* > *DBT*^s^ females showed a large proportion of individuals with a period of ~ 24 h, reflecting the pace of the molecular clock in the rest of the clock network (Figs 4F, 4G, and S4H). Some *Pdf* > *DBT*^s^ males (~23%) and a smaller proportion of females (~6%) also presented complex rhythms (Table 4). The average period value of the second-period component (which has a lower power value) was ~ 24.07 ± 0.4 for the experimental males and 19.7 ± 0.4 for the experimental females. Neither male nor female control flies exhibited complex rhythms (Table 4). Among rhythmic flies, both *Pdf* > *DBT*^s^ males and females had lower rhythmic power than the controls (Fig 4H), with no difference between the sexes (Fig 4I). As a control, we expressed *DBT*^*s*^ via *Clk856-Gal4* which is expressed in most clock neurons (S4A Fig). Speeding up the molecular clock in most clock neurons significantly advanced both the morning and evening activity peaks of both males and females (S4B–S4D Fig and S2 Table). There were no significant differences between experimental males and females in the percentage of rhythmic flies, rhythmic power, or shortening of FRPs following DBT expression in all clock neurons (S4E–S4G Fig). We also compared *per*^01^ mutant males and females, and our results show that both are nearly completely arrhythmic (S4I Fig).

**Table 4:** Table representing the % rhythmicity, % complex rhythms, free-running period, and rhythmic power of *control and Pdf > DBT*^*s*^ males and females.

*Pdf > DBT* ^*s*^					
Genotype	*n*	% Rhythmicity ± SEM	% Complex rhythms ± SEM	Free-running period ± SEM	Rhythmic power ± SEM
*Pdf-Gal4* (male)	90	95.86 ± 2.73	0	24.3 ± 0.03	111.8 ± 5.58
*UAS DBT*^*s*^ (male)	108	96.79 ± 2.21	0	23.9 ± 0.01	134.8 ± 4.44
*Pdf > DBT*^*s*^ (male)	93	69.87 ± 6.89^***#^	23.73 ± 5.51^***^	18.9 ± 0.14^***^	49.48 ± 3.54^***^
*Pdf-Gal4* (female)	66	93.56 ± 4.02	0	24.4 ± 0.03	122 ± 6.76
*UAS DBT*^*s*^ (female)	83	89.45 ± 2.74	0	24.2 ± 0.03	89.7 ± 5.19
*Pdf > DBT*^*s*^(female)	70	38.41 ± 2.74^***#^	5.54 ± 3.45	22.5 ± 0.58	24.8 ± 3.55^***^

* indicates that the experimental genotypes are significantly different from their respective control flies of the same sex. # indicates that experimental males and females are significantly different from each other. *p < 0.05, **p < 0.01, ***p < 0.001.

These results support the notion that M-cells are more dominant in the male than in the female circadian network. Previous studies have shown that blocking synaptic neurotransmission by expressing the tetanus toxin light chain (TeTxLC) in small and large LN_v_s affects male activity rhythms, likely in a *Pdf*-independent manner [58–60]. We analyzed male and female *Pdf* > TeTxLC flies and found that neither sex significantly changed the ability to maintain rhythmicity under free-running conditions (S5A and S5B Fig, and S3 Table). Both sexes significantly lengthened the FRP, but the effect was more pronounced in males (S5C and S5D Fig). Rhythmic power was not affected in experimental flies of either sex (S5E Fig). These results are consistent with previous studies which show that TeTxLC expression in *Pdf*+ neurons lead to behavioral phenotypes that are different from those of loss of *Pdf*, and suggest that manipulations of neuronal activity of the *Pdf*-expressing neurons have a more pronounced effect in male FRP.

**Fig 5:**
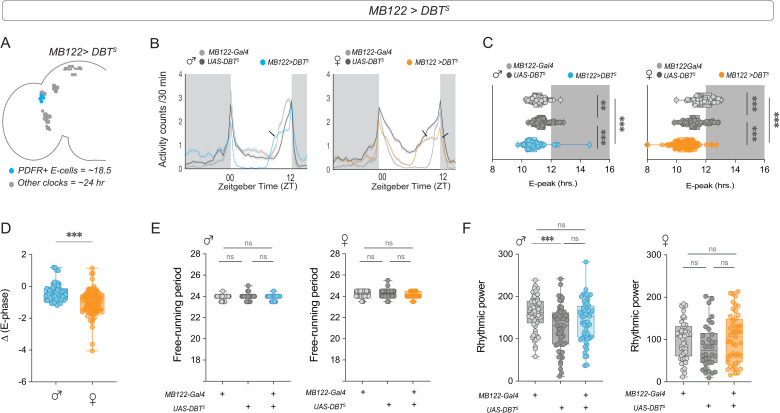
Speeding up the clock in an E-cell subset leads to a more advanced evening peak in females. **(A)** Depiction of the adult *Drosophila* brain hemisphere indicating the clock cell subsets (colored) having a faster running molecular clock. **(B)** Average activity plots of control (*MB122B-Gal4*) and (*UAS-DBT*^*s*^) and experimental (*MB122B > DBT*^*s*^) flies are plotted for males (left) and females (right). The plots are averaged over flies and days for a period of three days under LD 12:12. **(C)** Phase of the evening peak of activity under LD 12:12 for controls (*MB122B-Gal4*) and (*UAS-DBT*^*s*^) and experimental (*MB122B > DBT*^*s*^) males (left, *n* = 92 (*MB122B-Gal4*), *n* = 93 (*UAS-DBT*^*s*^), *n* = 93 (*MB122B > DBT*^*s*^)) and females (right, *n* = 85 (*MB122B-Gal4*), *n* = 87 (*UAS-DBT*^*s*^), *n* = 89 (*MB122B > DBT*^*s*^)). **(D)** The differences in the phase of the E peak between experimental males and females and their respective controls are plotted. **(E)** Free-running period of control (*MB122B-Gal4* and *UAS-DBT*^*s*^) and experimental (*MB122B > DBT*^*s*^) males (left) and females (right) calculated via the chi-squared periodogram are plotted. **(F)** Rhythmic power of control (*MB122B-Gal4 and UAS-DBT*^*s*^) and experimental (*MB122B*^* *^*> DBT*^*s*^) males (left) and females (right) calculated via the chi-squared periodogram are plotted. Statistical comparisons were performed between the control and experimental flies of both sexes using the Kruskal–Wallis test followed by Dunn’s multiple comparisons test for panels 5C, 5E, and 5F and Mann–Whitney *U* test for Fig 5D. The box plots extend from the 25th to 75th percentile, with whiskers extending from the smallest to the largest value, and each point represents data from a single fly. Combined data from at least three replicate experiments are plotted. **p < 0.01, ***p < 0.001.

We next asked whether changing the pace of the clock in LN_d_s (E-cells) via *DBT*^*S*^ expression also had sexually dimorphic effects on behavior. These cells can be subdivided into at least three different clusters on the basis of their anatomy [8], physiology [56], transcriptomic profiles [15], and connectivity patterns [17]. The PDFR-expressing E1 and E2 clusters have been shown to regulate evening activity under LD [61,62] and to be able to maintain free-running activity rhythms in the absence of a functional clock in M cells [63,64], whereas the behavioral role of the E3 cluster remains unknown. We used the MB122-B split-gal4 driver to target the E1 and E2 subsets (Fig 5A) and found that while expressing *DBT*^*S*^ in this group of evening cells significantly advanced the phase of the E-peak in experimental flies of both sexes (Fig 5B and 5C), the effect was more pronounced in females (Fig 5D). Speeding up of clocks in the E1 + E2 LN_d_s did not significantly alter the FRP or rhythmic power of experimental flies of either sex (Fig 5E and 5F). M-cells have been shown to be the dominant oscillators in DD and to regulate rhythm properties such as persistence and the FRP of endogenous locomotor rhythms to a large extent [18,22,23,56], although manipulations of other clock cells can affect rhythm properties to some extent [56,65]. Thus, speeding up the clock in the PDFR^+^ E1 and E2 clusters leads to similar behavioral phenotypes in males and females under free-running conditions. Taken together, these results indicate that the relative influence of the M and E subsets of clock neurons are sexually dimorphic.

## Discussion

The critical importance of considering sex as a biological variable has gained increasing recognition in biomedical research [66,67]. Bias toward male subjects is particularly prevalent in neuroscience, with single-sex studies using male animals outnumbering those using female animals at a ratio of 5.5:1 [68]. This disparity extends to chronobiology, resulting in a limited understanding of how sex affects circadian organization in the nervous system. However, work from several laboratories has revealed sexual dimorphism within the SCN and in its input and output pathways [1,69]. Sex differences in SCN morphology have been described in both animal models and humans, and sex differences in SCN electrical activity and steroid hormone receptors have also been reported (reviewed in [2]). Notably, sex differences in the number of SCN neurons that express the neuropeptide vasoactive intestinal polypeptide (VIP) and in *Vip* mRNA *e*xpression have been reported (reviewed in [2]). The roles of the mammalian VIP and the *Drosophila* PDF in circadian physiology are highly similar, although neither these peptides nor their receptors are sequence orthologs [70].

Several studies have shown the importance of PDF in generating coherent rhythms of ~ 24-h periodicity. Here, we report that females lacking *Pdf* or its receptor *PdfR* are more likely to maintain consolidated activity–rest behavior than males. This could be because of sex differences in PDF signaling mechanisms, PDFR expression, or the influence of other clock neurons within the network. In males, other neuropeptides are known to act in concert with PDF to maintain consolidated rhythms in the network, although none of them have as profound an effect as PDF in regulating activity-rest rhythms in DD [49,71]. Single mutants of DH31 and CCHamide1 do not affect activity rhythms by themselves, but the double mutants of these neuropeptides along with *Pdf*^*01*^ (*DH31*^*01*^*/Pdf*^*01*^ and *Pdf*^*01*^/*CCHa*^*SK8*^) are almost completely arrhythmic, suggesting that these neuropeptides act hierarchically in the network, with PDF being at the top of that hierarchy [49,71]. The importance of PDF relative to other peptides released by clock neurons may also be sexually dimorphic.

Although CRISPR manipulation was only partially effective at eliminating PDF expression, it nevertheless produced phenotypes reminiscent of those produced by the *Pdf*^*01*^ mutation. We observed faint staining in the dorsal projections of at least one s-LN_v_ in at least one hemisphere in most brains, and PDF staining in a single s-LN_v_ projection reaching the dorsal brain has been shown to be sufficient for behavioral rhythms [72]. Experimental flies in which *Pdf* was knocked out starting at the onset of promoter expression early in development showed extensive misrouting of their dorsal termini, similar to what has been reported for *Pdf*^*01*^ males [54]. Instances of s-LN_v_ misrouting have also been observed in other core clock mutants, such as *per*^*01*^ and *tim*^*01*^ [73] and *cyc*^*01*^ [74]. No correlation between misrouting and behavioral phenotypes was found by others for *Pdf*^*01*^ males [54]. Importantly, *Pdf > Pdfg;Cas9* manipulation recapitulates the sexually dimorphic circadian phenotypes of *Pdf*^*01*^ mutants: a larger fraction of females are rhythmic, and females exhibit greater rhythm power.

In males, changing the speed of the M cell clock leads to phase changes in the morning peak under LD [61]. To determine whether M-cell manipulations also have sexually dimorphic effects on behavior, we sped up the molecular clock by expressing the *doubletime short* allele (*DBT*^*s*^). Surprisingly, our results revealed that speeding up the clock of M-cells advances the phase of the morning peak in males, whereas the female morning peak phase is not affected. These results support previous studies conducted in males on the role of M-cells in regulating the morning peak of activity [18,23,61] and suggest that M-cells are unable to regulate the phase of morning activity in the same way in females. In DD, males had largely coherent short-term rhythms, and the majority (65%) were rhythmic. In contrast, only 40% of the females were rhythmic, and their period showed a bimodal distribution. These findings further support the notion that M-cells are less dominant than the circadian clock network in females. A possible explanation for this is that other clock neurons are able to “resist” their influence, and the conflict between the fast-paced M cell clock and the ~ 24 clock other clock neurons is what leads to greater arrhythmicity in females.

The expression of a TeTxLC construct in flies blocks neurotransmission by binding and cleaving the synaptic protein Synaptobrevin [75]. Expressing tetanus toxin in LN_v_s did not result in a reduction in rhythmic power, but it lengthened the male FRP, as reported in previous studies [59,60,76]. The behavioral phenotypes resulting from the blockade of synaptic transmission differ from those resulting from the loss of PDF [20] or the ablation of *Pdf-*expressing neurons [23], possibly because tetanus toxin affects classical transmission and not the dense core vesicle-mediated release of neuropeptides such as PDF [77]. Abrogating the dorsal termini of the small LN_v_s, where most of the output synapses are found [17,77], does not result in behavioral phenotypes similar to those of *Pdf*^01^ under either LD or DD [24]. Our results show that blocking synaptobrevin-dependent synaptic transmission in M-cells does not affect rhythmicity but rather lengthens the FRP. The period lengthening is more pronounced in males, supporting the notion that M-cells have a greater influence on the circadian network in males.

*Cryptochrome* and *PdfR*-expressing clusters of evening cells—the sNPF-expressing E1 cluster and the ITP-expressing E2 cluster [78]—have roles in setting the phase of the E-peak under LD and sustaining behavioral rhythms in the absence of a functional molecular clock in M-cells [63,64]. To test whether these cells have a differential influence on the network in males and females, we expressed *DBT*^S^ under a driver that is expressed specifically in the E1 and E2 subsets of LN_d_s. Our results showed that speeding up the clocks in the E1 + E2 clusters resulted in a phase advance in the evening peak of activity in both sexes, but the effect was more pronounced in females. A possible reason for the behavioral differences observed between the sexes could be redundancy in females, such that the network is not as dependent on PDF or M-cells for timekeeping. This finding suggests that the female network could have a more distributed mode of timekeeping throughout the circadian clock network.

Across species, sex differences in the circadian timing system are largely related to the regulation of reproduction-related behaviors. In mammals, the SCN determines the timing of the release of reproductive hormones and influences the timing of mating (reviewed in [69]) and aggression [79]. In *Drosophila*, the circadian clock controls the timing of sex-specific and sexually dimorphic behaviors, such as male courtship [80] and female sexual receptivity [81] and egg laying [82]. This regulation of rhythmic behaviors requires connectivity between clock neurons and downstream sex-specific circuits. For example, the DN1_p_ cluster, which has been shown to be more active in males [38], is functionally connected to the male-specific *fru*-expressing P1 neurons that regulate male courtship [39]. In females, Allatostatin C-producing DN1_p_s have been shown to connect to downstream targets to control rhythms in oogenesis [83], and the Janelia female hemibrain connectome revealed that the LN_d_s form connections with the *doublesex*-expressing PC1 cluster [17]. Our data suggest that the relative hierarchy of circadian oscillators is sexually dimorphic, with a less dominant M oscillator in females. Interestingly, when males are exposed to constant light (LL), the prevalence of the morning oscillator decreases, while the evening oscillator becomes more dominant [84–86]. One proposed mechanism for this shift in the hierarchy of circadian oscillators involves GW182, which influences the circadian neural network’s response to light and modulates the level of PDFR signaling [87]. If the evening oscillator is more dominant in the female circadian network, it is possible that males and females respond differently to constant light, at least under specific light intensities. The existence of sex differences in the hierarchy of circadian oscillators may serve an adaptive purpose, ensuring the precise timing of essential female-specific behaviors crucial for reproductive fitness, such as sexual receptivity and egg laying.

## Materials and methods

### Fly lines and rearing

All the genotypes were reared on corn syrup soy media (Archon Scientific; Durham, NC) under LD 12:12 cycles at 25 °C unless specified otherwise (see figure legends for details). The fly lines used in this study were *Canton-S*, *w*^*1118*^, *Pdf*^*01*^, *PdfR*^*01*^, *Pdf-RFP*, *Pdf-Gal4; tub-Gal80*^*ts*^, UAS-*Cas9;* UAS*-Pdfg*, *Pdf-Gal4*, UAS*-DBT*^*s*^, *UAS TeTxLC*, *Clk856Gal4*, *s-LNvGal4*, and *MB122B-Gal4.* See the fly lines and reagents table below for more details. All experiments were conducted with virgin females, as mating affects female rhythm strength [48]. We employed a *Pdf*^*01*^ mutant line outcrossed in the *w*^*1118*^ background. See Table 5 for details about fly lines.

**Table 5:** Fly lines and reagents.

REAGENT or RESOURCE	SOURCE	IDENTIFIER
**Experimental Models: Organisms/Strains**		
*Canton-S*	Bloomington *Drosophila* Stock Center	BDSC 64349
*w* ^*1118*^ *;+;+*	Bloomington *Drosophila* Stock Center	BDSC 3605
*yw;Pdf-RFP,Pdf-Gal4;Tub-gal80* ^*ts*^	Justin. Blau, NYU	
*w;;Pdf* ^*01*^	Paul Taghert, Wash U Med. School	
*w PdfR* ^*01*^ *;;*	Paul Taghert, Wash U Med. School	
*w; Pdf-gal4; +*	Paul Taghert, Wash U Med. School	
*;UAS Cas9; UAS pdfg*	Michael Rosbash, Brandeis University	BDSC 99650 (pdfg) and BDSC 58985 (Cas9)
*w; + ;DBT* ^*s*^	Jeffrey Price, University of Missouri	
*w; MB122B-Gal4; +*	Gerry Rubin, HHMI Janelia Research Campus	
*w; Clk856-Gal4; +*	Orie Shafer, CUNY ASRC	
*s-LNv Gal4*	Gerry Rubin, Janelia Research Campus	SS00681-Gal4
*w; UAS TeTxLC; +*	Cahir O’Kane, University of Cambridge	
**Antibodies**		
Rabbi anti-RFP (1:2000)	Rockland	#600-401-379-RTU
Mouse anti-PDF (1:3000)	Developmental Studies Hybridoma Bank	
Anti-rabbit Alexa-568 (1:3000)	Thermo Fisher	A11036
Anti-mouse Alexa-488 (1:3000)	Thermo Fisher	A11029
**Software**		
Fiji	http://fiji.sc	RRID: SCR_002285
MATLAB R2022b	MathWorks, Natick	RRID: SCR_001622
GraphPad Prism 9.0	GraphPad Software	RRID: SCR_002798
DAM FileScan	Trikinetics	
ClockLab	Actimetrics	RRID:SCR_014309
**Chemicals, Peptides, and Recombinant Proteins**		
Vectashield Mounting Medium	Vector Laboratories	#H-1000-10
Premix PBS Buffer (10x)	Sigma–Aldrich	Cat# 11666789001
2% Paraformaldehyde (PFA)	Sigma–Aldrich	47608-250ML-F
Triton-X-100	Bio Basic	CAS#9002-93-1
Schneider’s *Drosophila* Medium (S2)	Thermo Fisher	21720024
**Other**		
DAM2 *Drosophila* Activity Monitors	Trikinetics	
DAM Drosophila Environmental Monitors	Trikinetics	

### Activity recording and analysis

Individual male and virgin female flies (3–5 days old) were housed in glass locomotor tubes containing 2% agar–4% sucrose food on one end and yarn on the other end. Locomotor activity was recorded using Drosophila activity monitors (DAM, Trikinetics, Waltham, United States of America). The experiments were conducted in Tritech or Percival incubators under controlled light and temperature conditions. Flies were entrained to 12:12 LD cycles for at least 5 days and then transferred to constant darkness (DD) for at least 7 days at a constant temperature of 25 °C unless otherwise specified (see figure legends for details). The raw data obtained from the DAM system were scanned and binned into activity counts of 15-min intervals via the DAM File scan. The data were analyzed via the CLOCKLAB software (Actimetrics, Wilmette, IL).

The values of period and rhythmic power were calculated for a period of 7 days via a chi-squared periodogram with a cutoff of *p = 0.01*. The rhythmic power for each designated rhythmic fly was determined by subtracting the chi-squared significance value from the power of the periodogram. Flies that did not exhibit a periodicity peak above the significance threshold were categorized as “arrhythmic,” and their period and rhythmic power were not included in the analysis. The values of the morning and evening peaks were calculated via PHASE software [88]. The total LD sleep values for all the genotypes were calculated for a period of 3 days (LD days 2–4) via the PHASE software. Representative actograms were generated via ClockLab, and activity plots were generated via PHASE. The period, rhythmic power, total sleep, and phase values of all the flies for a particular experimental genotype were compared against the background or parental controls via either the Mann–Whitney test or the Kruskal–Wallis ANOVA followed by the Dunn’s multiple comparisons test. The details of the statistical comparisons and the number of flies used in a given experiment are indicated in their respective figure legends. The number of rhythmic flies of the experimental genotype was compared against their respective background or parental controls via Fisher’s exact test. All the statistical analyses were performed via GraphPad Prism 9.0.

If both the experimental males and females were significantly different from their respective control flies of the same sex, the extent of sex differences were calculated by subtracting the average values of the control from each individual experimental value. These differences were then directly compared between males and females using the appropriate statistical analysis (the test used in each case is mentioned in the figure legends for the respective figures). In case of experiments with two parental controls, the average value to calculate the difference would be the average of the Gal4 and UAS control genotypes.

### Immunohistochemistry

The brains of adult male or female flies were dissected in ice-cold Schneider’s insect media (S2) and fixed immediately after dissection in 2% paraformaldehyde (PFA) in S2 media for 30 min at room temperature. The fixed brains were washed (three washes of 10 min each) with 0.3% phosphate-buffered saline-Triton X 100 (PBS-TX) and then treated with blocking solution (5% normal goat serum made in 0.3% PBS-TX) for 1 h at room temperature. The brains were then incubated with primary antibodies at 4 °C for 24 h. The primary antibodies used were anti-PDF (mouse, 1:3000, C7, DSHB) and anti-RFP (rabbit, 1:2,000, Rockland Immunochemicals). After incubation, the brains were subjected to six washes with 0.3% PBS-TX and incubated with Alexa Fluor-conjugated secondary antibodies overnight at 4 °C. The following secondary antibodies were used: goat anti-mouse 488 (1:3,000, Invitrogen) and goat anti-rabbit 568 (1:3,000, Invitrogen). After incubation, the brain samples were washed six times with 0.3% PBS-TX, cleaned and mounted on a clean glass slide using Vectashield mounting media.

### Image acquisition and analysis

The brains were imaged via a confocal microscope (Olympus FV3000) with an Olympus UPLanXApo 20× or 40× objective. Image analysis was performed via Fiji software [89]. In the samples, small and large LN_v_s were classified on the basis of their anatomical locations and expression of the PDF. PDF intensities in these cells were measured by selecting the slice of the Z-stack that showed the maximum intensity, drawing a region of interest (ROI) around the cells, and measuring their intensities. Three to four separate background values were also measured around each cell, and the final intensity was taken as the difference between the cell intensity and the average background.

For quantification of the PDF in the dorsal projections, a rectangular box was drawn as the ROI starting from the point where the PDF projection turns into the dorsal brain, and the intensity is measured. Three to four background values were also measured around the projection. The intensity values obtained from both hemispheres for each cell type for each brain were averaged and used for statistical analysis. PDF intensity from the s-LN_v_ was compared between the experimental and control genotypes via a Mann–Whitney test. To estimate different aspects of rhythmicity in PDF oscillations in the dorsal termini of s-LN_v_ in males and females, we used a COSINOR-based curve-fitting method [Cornelissen, 2014]. COSINOR analysis was implemented via the CAT Cosinor function from the CATkit package written for R [90].

## Supporting information

S1 Fig PDF rhythmic accumulation is similar in males and females (related to Fig 1). **(A)** Representative confocal images of control (*w*^*1118*^) (top) and experimental (*Pdf*^*01*^) (bottom) male and female flies stained with the PDF antibody. **(B)** Scatterplots of PDF staining intensities of the s-LN_v_ dorsal projections of both male and female flies plotted at different time points over a 24-h cycle on DD day 3. Each dot represents the mean PDF intensity value averaged over both hemispheres of one brain. The cyan and pink lines are the best-fit cosine curves from the parameters that were extracted via COSINOR analysis. See Table 2 for more details. **(C)** Amplitude values of PDF oscillation obtained from COSINOR curve fits are plotted for male and female flies. The error bars are 95% CI values calculated from the standard error obtained from COSINOR analysis. The overlapping error bars indicate that the amplitude values of males and females are not significantly different. *n* > 7 brain samples/time point. **(D)** Nuclear RFP intensity values are plotted for the sLN_v_s of *Pdf > nls-mCherr*y males (*n* = 14 brains) and females (*n* = 10 brains).
(EPS)

S2 Fig CRISPR-Cas9-mediated *Pdf* mutagenesis has similar effects on male and female sleep (Related to Fig 3). **(A)** Average sleep plots under LD 12:12 of control (*Pdf-RFP, Pdf-Gal4; tub-Gal80*^*ts*^ *and UAS Cas9; Pdf-g*) and experimental (*Pdf-RFP, Pdf-Gal4; tub-Gal80*^*ts *^*> Cas9; Pdf-g*) males (left) and females (right) are plotted. The plots are averaged over flies and days for a period of 3 days under LD 12:12. The Gal4 and UAS controls are light and dark gray traces, and the experimental males and females are blue and magenta traces respectively. **(B)** Total sleep values under LD conditions are plotted for control (*Pdf-RFP, Pdf-Gal4; tub-Gal80*^*ts*^ *and UAS Cas9; Pdf-g*) and experimental (*Pdf-RFP, Pdf-Gal4; tub-Gal80*^*ts *^*> Cas9; Pdf-g*) males (left) and females (right). **(C)** Average sleep plots of flies over eight days in DD are plotted for male and female control (*Pdf-RFP, Pdf-Gal4; tub-Gal80*^*ts*^ *and UAS Cas9; Pdf-g*) and experimental (*Pdf-RFP, Pdf-Gal4; tub-Gal80*^*ts *^*> Cas9; Pdf-g*) flies. The Gal4 and UAS controls are light and dark gray traces, and the experimental males and females are blue and magenta traces respectively. **(D)** Total sleep values under DD 1–8 are plotted for control (*Pdf-RFP, Pdf-Gal4; tub-Gal80*^*ts*^ *and UAS Cas9; Pdf-g*) and experimental (*Pdf-RFP, Pdf-Gal4; tub-Gal80*^*ts *^*> Cas9; Pdf-g*) males (left) and females (right). **(E)** The differences in the total sleep values of experimental males and females from their respective controls are plotted for DD 1–8. **(F)** Total sleep values for DD day1 are plotted for control (*Pdf-RFP, Pdf-Gal4; tub-Gal80*^*ts*^ *and UAS Cas9; Pdf-g*) and experimental (*Pdf-RFP, Pdf-Gal4; tub-Gal80*^*ts *^*> Cas9; Pdf-g*) males (left) and females (right). **(G)** Activity/waking minute for DD day1 are plotted for control (*Pdf-RFP, Pdf-Gal4; tub-Gal80*^*ts*^ *and UAS Cas9; Pdf-g*) and experimental (*Pdf-RFP, Pdf-Gal4; tub-Gal80*^*ts *^*> Cas9; Pdf-g*) males (left) and females (right). Statistical comparisons were performed between the control and experimental flies of both sexes using the Kruskal–Wallis test followed by Dunn’s multiple comparisons test for panels S2B and S2D and Mann–Whitney *U* test for Panel S2E. The box plots extend from the 25th to 75th percentile, with whiskers extending from the smallest to the largest value, and each point represents data from a single fly. Combined data from at least three replicate experiments are plotted. **p < 0.01, ***p < 0.001.
(EPS)

S3 Fig CRISPR-Cas9-mediated *Pdf* mutagenesis in small ventral lateral neurons has more pronounced effects on male circadian behavior. **(A)** Percentage of rhythmic flies are plotted for control (*s-LNv-Gal4*) and (*UAS Cas9; Pdf--g*) and experimental (*s-LNv > Cas9; Pdf--g*) males (*n* = 72 (*s-LNv-Gal4*), *n* = 62 (UAS *Cas9; Pdfg*), *n* = 73 (*s-LNv > Cas9; Pdfg*)) and females (right, *n* = 61 (*s-LNv-Gal4*), *n* = 56 (*UAS Cas9; Pdfg*), *n* = 73 (*s-LNv > Cas9; Pdfg*)) **(B)** Free-running period of control (*s-LNv-Gal4 and UAS Cas9; Pdf-g*) and experimental (*s-LNv*^* *^*> Cas9; Pdf-g*) males and females calculated via the Chi-squared periodogram are plotted. **(C)** Rhythmic power of control (*s-LNv-Gal4 and UAS Cas9; Pdf-g*) and experimental (*s-LNv > Cas9; Pdf-g*) males (left) and females (right) calculated via the chi-squared periodogram are plotted. **(D)** The differences in rhythmic power between experimental males and females and their respective controls are plotted. **(E)** Representative actograms of control (*s-LNv-Gal4*) and (*UAS Cas9; Pdfg*) and experimental (*s-LNv > Cas9; Pdfg*) males (left) and females (right) are plotted for 5 days of LD followed by 10 days of DD. Statistical comparisons were performed between the control and experimental flies of both sexes using the Kruskal–Wallis test followed by Dunn’s multiple comparisons test. Percentage of rhythmic flies were compared using Fisher’s exact test. The box plots extend from the 25th to 75th percentile, with whiskers extending from the smallest to the largest value, and each point represents data from a single fly. *p < 0.05, **p < 0.01, ***p < 0.001.
(EPS)

S4 Fig Speeding up the clock in all clock cells leads to a phase advance and shortening of the free-running period of activity rhythms (related to Figs 4 and 5). **(A)** Diagram of an adult *Drosophila* brain hemisphere indicating the clock cell subsets (blue) expressing DBT^s^. **(B)** Average activity plots of control (*Clk856-Gal4*) and (UAS-*DBT*^*s*^) and experimental (*Clk856 > DBT*^*s*^) flies are plotted for males (left) and females (right). The plots are averaged over flies and days for a period of three days under LD 12:12. **(C)** Phases of the morning peak of activity under LD 12:12 for controls (*Clk856-Gal4*) and (*UAS-DBT*^*s*^) and experimental (*Clk856 > DBT*^*s*^) males (left, *n* = 62 (*Clk856-Gal4*), *n* = 57 (*UAS--DBT*^*s*^), *n* = 60 (*Clk856 > DBT*^*s*^)) and females (right, *n* = 59 (*Clk856-Gal4*), *n* = 59 (*UAS--DBT*^*s*^), *n* = 50 (*Clk856 > DBT*^*s*^)) are plotted. **(D)** Phase of the evening peak of activity under LD 12:12 for controls (*Clk856--Gal4*) and (*UAS-DBT*^*s*^) and experimental (*Clk856 > DBT*^*s*^) males (left) and females (right) are plotted. **(E)** Percentage of rhythmic flies are plotted for control (*Clk856--Gal4*) and (*UAS-DBT*^*s*^) and experimental (*Clk856 > DBT*^*s*^) males (left) and females (right). The error bars represent the SEM values plotted across two replicate experiments. **(F)** Free-running periods of rhythmic flies calculated via the chi-squared period are plotted for controls (*Clk856--Gal4*) and (*UAS-DBT*^*s*^) and experimental (*Clk856 > DBT*^*s*^) males (left) and females (right). **(G)** Rhythmic power of flies calculated via the Chi-squared periodogram is plotted for control (*Clk856--Gal4*) and (*UAS-DBT*^*s*^) and experimental (*Clk856 > DBT*^*s*^) males (left) and females (right). **(H)** Representative actograms of *Pdf > dBTs* females showing free-running period values close to 24 h. **(I)** Percentage of rhythmic flies are plotted for 10 days in DD for control (Canton-S) and *Per*^*01*^ males and females. Statistical comparisons were performed between the control and experimental flies of both sexes via Kruskal–Wallis test followed by Dunn’s multiple comparisons test. Percentage of rhythmic flies were compared using Fisher’s exact test. The box plots extend from the 25th to 75th percentile, with whiskers extending from the smallest to the largest value, and each point represents data from a single fly. Combined data from at least two replicate experiments are plotted. *p < 0.05, **p < 0.01, ***p < 0.001.
(EPS)

S5 Fig Blocking neurotransmission in *Pdf*-expressing cells leads to more pronounced lengthening of the free-running period in males (Related to Fig 4). **(A)** Representative actograms of control *(Pdf-Gal4*) and (*UAS TeTxLC*) and experimental (*Pdf > TeTxLC*) males (left) and females (right) are plotted for five days of LD followed by eight days of DD. **(B)** Percentage of rhythmic flies are plotted for control (*Pdf-Gal4*), and (*UAS TeTxLC*), and experimental (*Pdf > TeTxLC*) males (left, *n* = 56 (*Pdf-Gal4*), *n* = 51 (UAS *TeTxLC*), *n* = 52 (*Pdf > TeTxLC*)) and females (right, *n* = 53 (*Pdf-Gal4*), *n* = 52 (*UAS TeTxLC*), *n* = 52 (*Pdf > TeTxLC*)). Error bars are SEM values plotted across two replicate experiments. **(C)** Free-running period of control (*Pdf-Gal4* and UAS *TeTxLC*) and experimental (*Pdf > TeTxLC*) males (left) and females (right) calculated using the Chi-squared Periodogram are plotted. **(D)** The difference in free-running period of experimental males and females from their respective controls are plotted. **(E)** Rhythmic power of control (*Pdf-Gal4* and *UAS TeTxLC*) and experimental (*Pdf > TeTxLC*) males (left) and females (right) calculated using the Chi-squared periodogram are plotted. Statistical comparisons were performed between the control and experimental flies for both sexes using the Kruskal–Wallis test followed by the Dunn’s multiple comparisons test for all panels except S6D where comparisons were made using the Mann–Whitney *U* test. Percentage of rhythmic flies were compared using Fisher’s exact test. The box plots extend from the 25th to 75th percentile, with whiskers extending from the smallest to the largest value, and each point representing data from a single fly. Combined data from at least two replicate experiments are plotted.
(EPS)

S1 Table Table representing the *n*, % rhythmicity, free-running period, and rhythmic power of *control and s-LNv > Cas9; Pdfg* males and females. ^*^ indicates that the experimental genotypes are significantly different from their respective control flies of the same sex. ^#^ indicates that experimental males and females are significantly different from each other. *p < 0.05, **p < 0.01, ***p < 0.001.
(DOCX)

S2 Table Table representing the *n*, % rhythmicity, free-running period, and rhythmic power of *control and Clk856 > dBTs* males and females. ^*^ indicates that the experimental genotypes are significantly different from their respective control flies of the same sex. ^#^ indicates that experimental males and females are significantly different from each other. * *p* < 0.05, ** *p* < 0.01, *** *p* < 0.001.
(DOCX)

S3 Table Table representing the *n*, % rhythmicity, free-running period, and rhythmic power of *control and Pdf > TeTxLC* males and females. ^*^ indicates that the experimental genotypes are significantly different from their respective control flies of the same sex. ^#^ indicates that experimental males and females are significantly different from each other. * *p* < 0.05, ** *p* < 0.01, *** *p* < 0.001.
(DOCX)

## Abbreviations

DAM: Drosophila activity monitors
DBT: doubletime
FRP: free-running period
LDs: light–dark cycles
LN_d_: dorsolateral neurons
LNs: lateral neurons
LN_v_: ventrolateral neurons
LN_v_s: ventral lateral neurons
LPNs: lateral posterior neurons
M-cells: morning cells
PBS-TX: phosphate-buffered saline-Triton X 100
PDF: pigment-dispersing factor
PFA: paraformaldehyde
ROI: region of interest
SCN: suprachiasmatic nuclei
s-LN_v_s: small ventral lateral neurons
TeTxLC: tetanus toxin light chain
VIP: vasoactive intestinal polypeptide
