# Supplementary material for: Circadian rhythms are more resilient to pacemaker neuron disruption in female Drosophila
Source: PLoS Biol. 2025 May 6;23(5):e3003146. doi: 10.1371/journal.pbio.3003146 (PMC12080924; doi:10.1371/journal.pbio.3003146)
Supplement: S1 Table — * indicates that the experimental genotypes are significantly different from their respective control flies of the same sex. # indicates that experimental males and females are significantly different from each other. *p < 0.05, **p < 0.01, ***p < 0.001. (DOCX) [file pbio.3003146.s006.docx]

**Supplementary Table S1**

*s-LNv > Cas9; Pdfg*

| Genotype | n | % Rhythmicity ± SEM | Free-running period ± SEM | Rhythmic power ± SEM |
| --- | --- | --- | --- | --- |
| *s-LNv-Gal4* (male) | 72 | 95.10 ± 2.48 | 24.46 ± 0.04 | 90.96 ± 4.92 |
| *UAS Cas9; pdfg* (male) | 62 | 100 ± 0.00 | 23.72 ± 0.03 | 137.98 ± 5.91 |
| *s-LNv > cas9; pdfg* (male) | 73 | 29.37 ± 2.21^***#^ | 21.95 ± 0.15^***^ | 21.31 ± 2.18^***^ |
| *sLNv-Gal4* (female) | 61 | 88.86 ± 11.13 | 24.75 ± 0.04 | 98.37 ± 7.60 |
| *UAS Cas9; Pdfg* (female) | 56 | 91.30 ± 4.41 | 24.19 ± 0.04 | 81.70 ± 7.54 |
| *sLNv > Cas9; Pdfg* (female) | 73 | 56.73 ± 10.54^***#^ | 22.44 ± 0.15^***^ | 28.44 ± 3.98^***^ |
