# Supplementary material for: Circadian rhythms are more resilient to pacemaker neuron disruption in female Drosophila
Source: PLoS Biol. 2025 May 6;23(5):e3003146. doi: 10.1371/journal.pbio.3003146 (PMC12080924; doi:10.1371/journal.pbio.3003146)
Supplement: S2 Table — * indicates that the experimental genotypes are significantly different from their respective control flies of the same sex. # indicates that experimental males and females are significantly different from each other. * p < 0.05, ** p < 0.01, *** p < 0.001. (DOCX) [file pbio.3003146.s007.docx]

**Supplementary Table S2**

*Clk856 > dBTs*

| Genotype | *n* | % Rhythmicity ± SEM | Free-running period ± SEM | Rhythmic power ± SEM | M-phase ± SEM | E-phase ± SEM |
| --- | --- | --- | --- | --- | --- | --- |
| *Clk856-Gal4* (male) | 83 | 97.73 ± 1.13 | 23.75 ± 0.03 | 95.27 ± 5.07 | -0.04 ± 0.06 | 11.90 ± 0.08 |
| *UAS dBTs* (male) | 63 | 98.60 ± 1.40 | 23.75 ± 0.06 | 92.19 ± 4.99 | -0.21 ± 0.16 | 11.74 ± 0.09 |
| *Clk856 > dBTs* (male) | 86 | 100 ± 0.00 | 17.99 ± 0.03^***^ | 104.87 ± 5.76 | -1.11 ± 0.16^***^ | 8.92 ± 0.20^***^ |
| *Clk856-Gal4* (female) | 75 | 69.87 ± 4.85 | 23.88 ± 0.04 | 51.01 ± 4.48 | 0.94 ± 0.07 | 11.66 ± 0.09 |
| *UAS dBTs* (female) | 49 | 74.23 ± 12.39 | 24.01 ± 0.06 | 66.46 ± 6.49 | 1.27 ± 0.07 | 11.51 ± 0.12 |
| *Clk856 > dBTs* (female) | 65 | 69.68 ± 5.99 | 17.94 ± 0.04^***^ | 59.02 ± 6.25 | -0.42 ± 0.1^***^ | 8.65 ± 0.21^***^ |
