# Supplementary material for: Circadian rhythms are more resilient to pacemaker neuron disruption in female Drosophila
Source: PLoS Biol. 2025 May 6;23(5):e3003146. doi: 10.1371/journal.pbio.3003146 (PMC12080924; doi:10.1371/journal.pbio.3003146)
Supplement: S3 Table — * indicates that the experimental genotypes are significantly different from their respective control flies of the same sex. # indicates that experimental males and females are significantly different from each other. * p < 0.05, ** p < 0.01, *** p < 0.001. (DOCX) [file pbio.3003146.s008.docx]

**Supplementary Table S3**

*Pdf > TeTxLC*

| Genotype | n | % Rhythmicity ± SEM | Free-running period ± SEM | Rhythmic power ± SEM |
| --- | --- | --- | --- | --- |
| *Pdf-Gal4* (male) | 56 | 92.15 ± 4.65 | 24.57 ± 0.03 | 105.32 ± 7.40 |
| *UAS TeTxLC* (male) | 51 | 95 ± 5.00 | 24.07 ± 0.04 | 90.92 ± 7.11 |
| *Pdf > TeTxLC* (male) | 52 | 88.1 ± 8.10^***#^ | 25.08 ± 0.05^***^ | 93.70 ± 7.99^***^ |
| *Pdf-Gal4* (female) | 53 | 90.60 ± 5.50 | 24.64 ± 0.04 | 104.90 ± 7.33 |
| *UAS TeTxLC* (female) | 52 | 88.40 ± 0.40 | 24.71 ± 0.05 | 103.59 ± 6.82 |
| *Pdf > TeTxLC*(female) | 52 | 87.85 ± 5.25^***#^ | 25.17 ± 0.07^***^ | 80.43 ± 7.20^***^ |
